# Crisis-ready responsible selves: National productions of the pandemic

**DOI:** 10.1177/13678779211066328

**Published:** 2022-07

**Authors:** Shani Orgad, Radha Sarma Hegde

**Affiliations:** 4905 London School of Economics and Political Science, UK; 5894 New York University, USA

**Keywords:** citizenship, Covid-19, gender, government campaigns, nationalism, neoliberal rationality, responsibilization

## Abstract

National governments have played a key role in constructing the Covid-19 pandemic through their communications. Drawing on thematic, discursive and visual analyses of Covid-19 campaigns from 12 national contexts, we show how the pandemic has presented governments with unique conditions for articulating and reinforcing nationalism and neoliberalism. The campaigns frame the pandemic as a force that brings the nation together and conjure up notions of national ‘solidarity lite’ while relentlessly authorizing the crisis-ready responsible citizen. In so doing, they reproduce neoliberal rationality by shifting the locus of responsibility from the state and social structures to the individual and re-inscribing gendered and classed notions of responsibility, care and citizenship. Mobilizing national neoliberal narratives enables governments to render the pandemic legible as a crisis while obscuring both the structural injustices that exacerbate the crisis and the structural changes required to address it.

The rapid spread and dire consequences of the Covid-19 pandemic have led nation states to spring into action to contain the virus, close borders, manage populations and recuperate economies. As citizens were being urged to mask, wash hands, bump elbows and socially distance to ‘flatten the curve’, the pandemic laid bare systemic failures of the state to provide infrastructures of equitable support. Many hoped the pandemic would present a moment which could significantly challenge the existing neoliberal order and patriarchal and racial capitalism (e.g. Chatzidakis et al., 2020; Lent, 2020; Women’s Budget Group, 2020). Yet, as we show in this article, across varying national contexts, the pandemic seems to have provided a fertile ground for the neoliberal state to revitalize its exhortations to citizens to comply with crisis-driven directives.

While the coronavirus has puzzled scientists by its newness, and while the pandemic has been described endlessly as ‘unprecedented’, the social and political roots and fallout of the crisis have been predictable. Indeed, as Butler (2020) writes, the rapidity with which radical inequality, nationalism, and capitalist exploitation find ways to reproduce and strengthen themselves within the pandemic should come as no surprise. As emerging research shows, pandemic protocols have provided a context for the rapid mediated reproduction of neoliberal logics and nationalism (Gill and Orgad, 2022; Sobande, 2020; Zou, 2021), or what might be called a ‘banal neoliberal nationalism’ (Orgad, 2012), with the individual citizen serving as the primary framework for making sense of and being responsible for managing the crisis. According to Brown (2015: 84),

The idea and practice of responsibilization — forcing the subject to become a responsible self-investor and self-provider — reconfigures the correct comportment of the subject from one naturally driven by satisfying interests to one forced to engage in a particular form of self-sustenance that meshes with the morality of the state and the health of the economy.

Indeed, as national governments have sought to handle the Covid-19 pandemic, emergent scripts mandating personal responsibility and self-care have redefined what it means to be a responsible citizen in the context of a viral crisis.

The pandemic spotlighted people's profound dependence on the state's support: from images of people in India lining up outside oxygen refilling centres, to reports about various countries’ lack of protective equipment for health and care workers, to redundancies, school and nursery closures which have had dramatically unequal impacts on people, and especially women, across the globe. Yet against this sobering background, since the beginning of the pandemic, governments have persistently centred the nation and promulgated vacuous messages of national solidarity, while relentlessly exhorting their citizens to act as independent and responsible subjects prepared to battle contagion. The consumer citizen has been anointed as the prime enforcer of hygiene, wielding a semblance of power to hold the virus at bay. It is this strategic reconfiguration of a global crisis as the responsibility of individuals within their nations, compounded by deep structural exclusions, to which we turn our critical attention in this article.

Crises are configured and defined through mediation, narratives and representation (Frosh and Pinchevski, 2009; Hay, 1996; Orgad, 2012). Drawing on theorizations that argue that crisis constitutes a critical point that demands that we see and hear things about social, political and economic life that we do not wish to hear (Dowling, 2021; Morin, 1984, cited in Wieviorka, 2012), in this article we argue that the pandemic constitutes a tipping point that ‘enables the diagnosis’ (Wieviorka, 2012: 96) of how national scripts of crisis keep the neoliberal order alive. More specifically, we focus our attention on Covid-19 national campaigns produced by governments – key cultural agencies in the production of global crises (Schwarz et al., 2016). We show how these campaigns’ productions of the pandemic are predicated on tactics that enable the reproduction of neoliberal rationality that relentlessly authorizes the crisis-ready responsible citizen against a backdrop of a vapid and amorphous collectivity. Capitalizing on the pandemic as a crisis that has foregrounded people's interdependence and the significance of community, these campaigns have centred and pumped-up national identity *and* individuals’ self-responsibility. Thus, against the promise that Covid-19 might constitute a ‘frame-breaking moment’, which could establish a sudden change in social normativity (drawing on Bilge, 2019: 111), we highlight how, as the pandemic unfolded, national government communications reproduced and cemented nationalism and neoliberal rationality concurrently. The pandemic presented governments across national contexts with a conducive moment to consolidate the notion of the national public as an atomized aggregate of responsible individuals.

In what follows we set out the specific questions for this study and our approach to addressing them. We then move to the empirical part of the article where we analyse examples of campaigns produced by national governments. In our conclusion we draw together the threads of the argument, reflecting on the ideological work the campaigns perform in leveraging the crisis to reproduce neoliberal logics of responsible citizenship and obscuring the structural injustices that underpin and exacerbate the crisis, as well as the structural changes that could alleviate it.

## Studying national Covid-19 crisis scripts

In this article we are interested in how national Covid-19 campaigns construct the pandemic as a crisis. More specifically, we ask: What cultural meanings do these campaigns generate about the pandemic as a crisis? How do they construct the nation in its relation to the world and what type of subjects do they authorize and idealize? And, crucially, what ideas, subjects and contexts do they render invisible? We show how various campaigns frame the pandemic as a national crisis whose overcoming is the responsibility of the individual citizen, who is idealized through the figure of the entrepreneurial warrior. National community, solidarity and bonding are valorized concurrently with a vapid and amorphous sense of collectivity and an atomized version of community, in which handling the crisis is all down to the individual.

Our discussion is based on an analysis of national Covid-19 campaigns produced by the governments of 12 countries: Argentina, Chile, Denmark, India, Israel, Japan, Kazakhstan, Mexico, South Korea, Spain, Switzerland and the UK. We focus on these governments’ communications at different points of the pandemic between March 2020 and May 2021 (the last month of our data collection), but not including any communication related to vaccinations.^1^ The sample from these 12 countries comprised of 43 texts, including billboards, outdoor and online posters, banners and slogans.^2^ We used a convenience sample: the selection of the countries was based on our access to, familiarity with and understanding of primary data sources, cultural contexts and languages, either directly (Radha Hegde: India; Shani Orgad: Israel and UK) or with the generous collaboration of our students and colleagues who are native speakers, and provided the contexts and translations for some of the campaigns and texts that we analysed. Their contribution is acknowledged at the end of the article. In some instances (e.g. in relation to Denmark's, Mexico's and Japan's campaigns), to further contextualize and corroborate our analysis, we consulted some press commentaries, tweets or blogs written in relation to specific national campaigns. We recognize that a convenience sample is not representative and cannot be generalized from. However, the fact that the examples in our sample were not selected according to predetermined criteria (except that they are all taken from national government campaigns) could be said to constitute an advantage, insofar as it highlights how across arguably very different national and cultural contexts as well as temporal stages of the pandemic, there are some broad and striking commonalities. Indeed, the primary objective of this article is to highlight how the figure of the responsibilized national citizen is constructed and authorized in similar ways in different national sites. Despite the variance in the structures of social life and political cultures of the 12 countries in our sample, the overwhelming turn to neoliberal rationality is striking, suggesting a global discursive regularity. It is this regularity of representation in national campaigns that we focus on in the sections that follow.

We first analysed each of the 12 countries’ campaign materials individually, using thematic, discursive and visual analyses. We paid particular attention to the visual aspect of national communications, since this is the aspect centred by the media we examine – namely billboards, outdoor and online posters and banners – and which played a central role in communicating the crisis to publics (Brennen et al., 2020; Kennedy, 2020). However, the global pathways of the virus invite a mode of critical looking at multiple national contexts *simultaneously*. Therefore, we next applied what we term ‘synchronous scanning’, examining thematic, discursive and visual similarities across the different cases. We found that while each of the campaigns responds to nation-specific aspects of the pandemic, there is a common narrative in all of them. This narrative maintains that the pandemic is fundamentally a local crisis, whose resolution relies on the actions of the individual, self-responsibilized gendered citizen, who is told to take care of herself and her community. In what follows we examine how this narrative of the Covid-19 crisis is constructed across the selected national campaigns. It is structured around three central tenets: (1) recentring the nation: solidarity lite; (2) the saviour citizen; and (3) the gendered model of the crisis.

## Recentring the nation: solidarity lite

The communications we examine are national campaigns and, as such, address national publics. However, one of the most striking aspects across all the examples in our sample is the leveraging of the pandemic to recentre the nation and reassert national identity and a glaring absence of any recognition of the borderless nature of the virus and a need for a concerted global effort to tackle the pandemic. While notions of global togetherness, unity and solidarity have circulated during the pandemic in some media (Sobande, 2020) – however hollow and disingenuous their meanings – they are entirely missing from the national campaigns we studied. Echoing Sarah Banet-Weiser’s (2012) observation about brand advertising in the aftermath of the 2008 financial crisis, rather than gesturing towards a global community that needs to unite in response to a global crisis, these national Covid-19 campaigns mobilized the crisis to recentre the nation and the individual's role in it, as a ‘way to reassert cultural control over an otherwise destabilizing crisis narrative’ (Banet-Weiser, 2012: 109).

**Figure 1.**
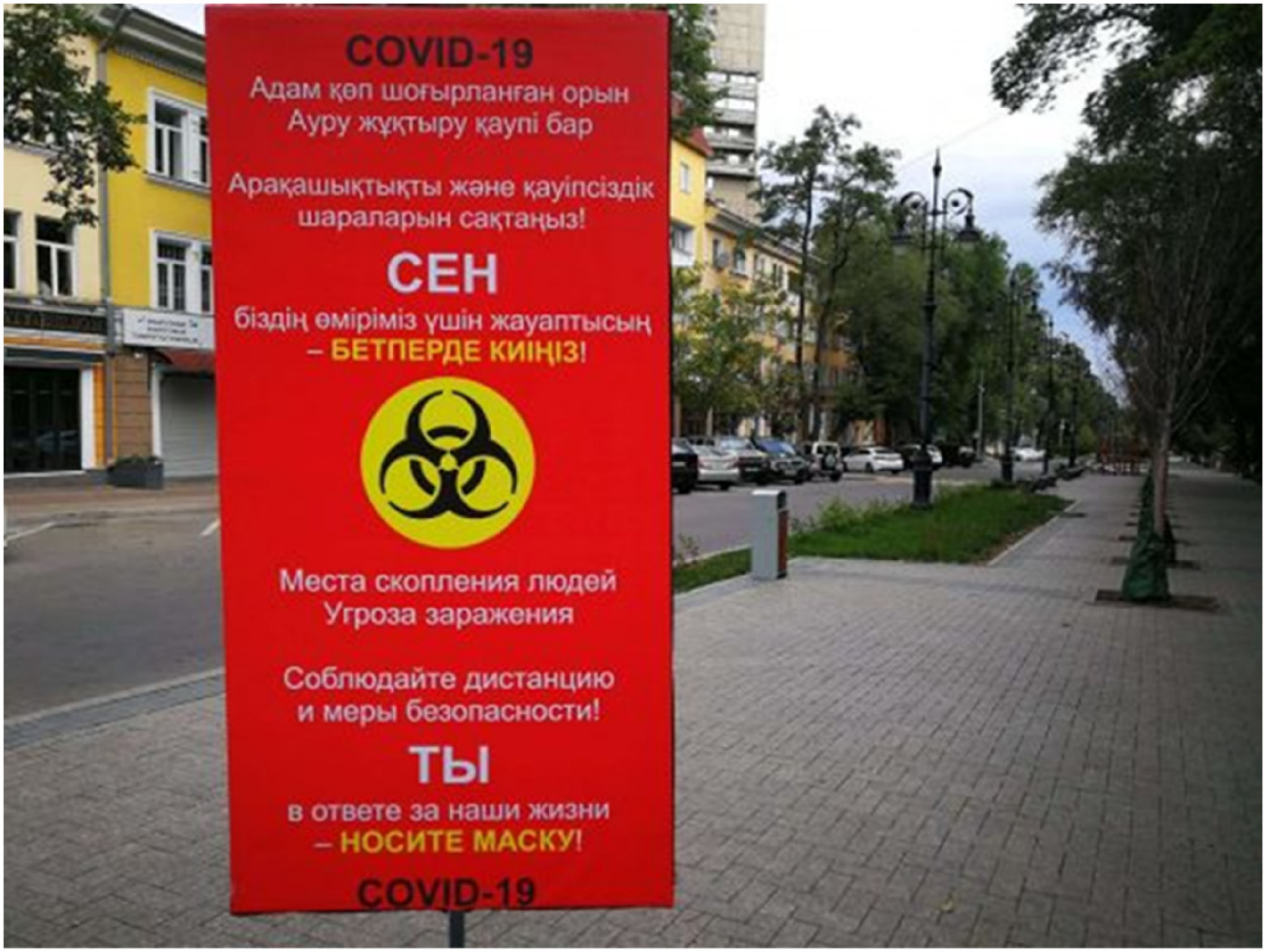
‘Crowded places. Risk of getting infected. Maintain social distancing and follow safety measures. YOU are responsible for our lives – wear a mask! COVID-19’ (Kazakhstan)

The reassertion and recentring of the nation are evident in several ways. First, campaigns draw on and appropriate familiar national aesthetics, discourses and traditions. For instance, the Japanese campaign employs *kyara*, the cartoon anthropomorphic characters that are ubiquitous in Japanese commercial marketing and PR (Occhi, 2010). They include, among others, a quarantine mascot, called Quaran, which was created by Japan's Ministry of Health, Labour and Welfare to promote the work of the Quarantine Information Office (Imada, 2020). Similarly, Kazakhstan's national campaign uses the colour red and warning symbols associated with radioactivity, which evoke past national public health risks, signifying a high-risk emergency (Figure 1).

Yet another example is the Israeli campaign, which employs a pun on the colloquial saying associated with Israeli culture, namely ‘Yihiye Beseder’, meaning ‘It will be fine’. The pun highlights the supposed choice citizens have between ‘We will be fine’ (‘Nihiye Be*seder*’) and ‘We will be in lockdown’ (‘Nihiye Be*seger*’), and the individual citizen's responsibility to ensure the first option – that the nation will be ‘fine’ rather than in lockdown (the second option). National traditions embodied by traditional gestures and customs are also used to communicate the crisis. For example, in the context of the current hyper-nationalist climate in India, the pandemic has been appropriated to make a pitch for authentic nationalism by evoking tradition and reclaiming traditional practices. In one of the Indian campaigns, the traditional namaste greeting is invoked as an alternative to the Western handshaking, resorting to tradition as the responsible and safe choice that citizens ought to make. Another campaign capitalizes on the Indian tradition of yoga, calling for citizens to ‘Combat Covid-19 with Yoga’ and ‘Be with yoga, be at home, stay safe, stay fit’ to build both the immunity and the optimism necessary in the fight against the pandemic.

Some campaigns go beyond appropriations of national discourses, symbols and traditions to explicitly invoke national memory, history, and a sense of national pride and patriotism. For example, in the Danish campaign, the word ‘*samfundssind*’ – a compound noun made up of ‘*samfund*’ (society) and ‘*sind*’ (mind) – was revived by Prime Minister Mette Frederiksen in March 2020, as she set out the country's response to the pandemic. Dating back to 1936, when the word ‘made an historical cameo in a call for solidarity […] at the outbreak of World War II’ (Johanson, 2020), the word was reintroduced by the Danish government to instil in the public an ethos of collective responsibility and community spirit. Similarly, in the Chilean campaign, references were made to the nation's historical ‘resilient spirit’, recouping a pristine past associated with glorious national achievements in global sports events and the nation's admirable responses to natural disasters.

More broadly, capitalizing on the touting of the virus as ‘the great equalizer’ – notoriously by Madonna in an Instagram message with a photo in her bathtub, and by politicians such as New York Governor Andrew Cuomo – national Covid-19 campaigns frame the pandemic as a force that brings the nation together. They conjure up notions of national social bonds, compassion, solidarity, unity, mutual support and collective care, reinforcing an imagined egalitarian national community. This framing can be seen in nearly all of the campaigns in our sample, for example, Chile's slogans ‘Let's *take care* of *each other*’ and ‘Taking care of each other is everyone's job’; Denmark's ‘Apart, to get together again’ and the use of ‘*samfundssind*’ (as discussed above); India's slogans ‘Our life is our responsibility’, ‘Save yourself, save others’, ‘I protect you, you protect me’; Israel's ‘Either *we’ll* be fine or *we’ll* be in lockdown’; Japan's ‘*Together we* stop the spread’; Kazakhstan's ‘You are responsible for *our* lives’ and ‘*Let*‘*s* get back to old ways of life’; Mexico's slogan ‘If you take care of yourself, *we* take care of *us all*’; Spain's urging of its citizens ‘We stop this together’; Switzerland's ‘This is how we protect ourselves’; and the UK's ‘Stay home, protect the *NHS* [National Health System], save *lives*’ and ‘We must keep on protecting each other’ (italics added).

However, not only does the emphasis on national togetherness and unity elide the crucial global dimension of the crisis and the interdependency of nations in responding to the pandemic, it also masks the persistent and worsening inequalities and disparities *within* nations. This is vividly exposed in the proliferating memes, spoofs and critiques that attack governments’ (and brands’) cynical disguising of inequalities under the veneer of ‘We’re all in this together.’ For example, responses to the UK government's slogans highlighted its occlusion of economic disparities in memes such as: ‘New government plan: stay alert, sacrifice the working class, save capitalism’ (10 May 2020)^3^ and a meme with a photo of Prince Charles and the text reading: ‘We’re all in this together. Except for those who enjoy massive privilege at public expense’ (weegingerdug.scot). Similarly, Indian citizens have been responding to Prime Minister Modi's speeches, taking his cautionary lines about appropriate Covid behaviour and turning them into jokes and memes on social media.

A proper exploration of such critical responses is beyond the scope of this article. However, we mention them to highlight how national campaigns, whose official purpose is to inform and educate citizens about risks and safety measures, concurrently perform an ideological work. More specifically, in a time where narratives that underscore social and economic inequalities and disparities are crucial in order to recognize and reinforce the need for interventions that redistribute access to services and resources (Milan et al., 2021), the national campaigns we studied have done precisely the opposite: they promoted narratives that ignored and masked inequalities and, instead, promulgated national solidarity and solidarity ‘lite’. Sobande (2020: 1034) critiques how brands’ rhetorical commitment to collectivity, and their framing of the pandemic as a unifying social force, helped ‘distract from their dubious treatment of employees, as well as their thirst for productivity and profit’. By the same token, the messages of national solidarity and unity promoted by the national campaigns in our sample occlude the crucial role that government responses to the pandemic (or their absence) played in perpetuating and exacerbating intersecting structural oppressions. The state's abdication of responsibility is further reinforced by the foregrounding of the logic of neoliberal citizenship and evocations of a very particular ideal citizen – a theme to which we turn next.

## The saviour citizen

The national bonding, community and solidarity that the national campaigns foreground are predicated on an atomized version of community, in which management of the crisis is down purely to the individual, and the public is implied to be an aggregate of individuals. This is vividly manifest in the campaigns’ visual features. Most of the campaigns use illustrations or photographs of faces wearing masks and socially distanced individual bodies to emphasize the instruction for social distancing and isolation. Of course, citizen response is a central feature of most crisis communications, especially in the case of pandemics, where the spread of diseases depends on human behaviour (Hyvärinen and Vos, 2016). However, crucially, these images also work to clear the state of any responsibility for managing the crisis. It is striking how there are barely any visual signifiers of the state and what *it* is doing to protect its people; rather, the focus is almost exclusively on what you (the individual) can do to protect yourself and others. Discursively, the use of directive illocutionary acts is prevalent across most of the campaigns – for example, ‘Stay home’ (Mexico, UK), ‘Take care of yourself’ (Mexico), ‘Wear a mask’ (Kazahhstan, South Korea), ‘Limit conversations during your meal’ (South Korea), ‘Save yourself, save others’ (India) – suggesting that the state's main, if not only responsibility, is to issue commands to its citizens about what to do and ensure that they obey them. Notably, there is an unrealistic and unjust levelling of class differences and a disregard for social differences in these directives, as if health care, digital devices and safe spaces are equally accessible to all.

**Figure 2.**
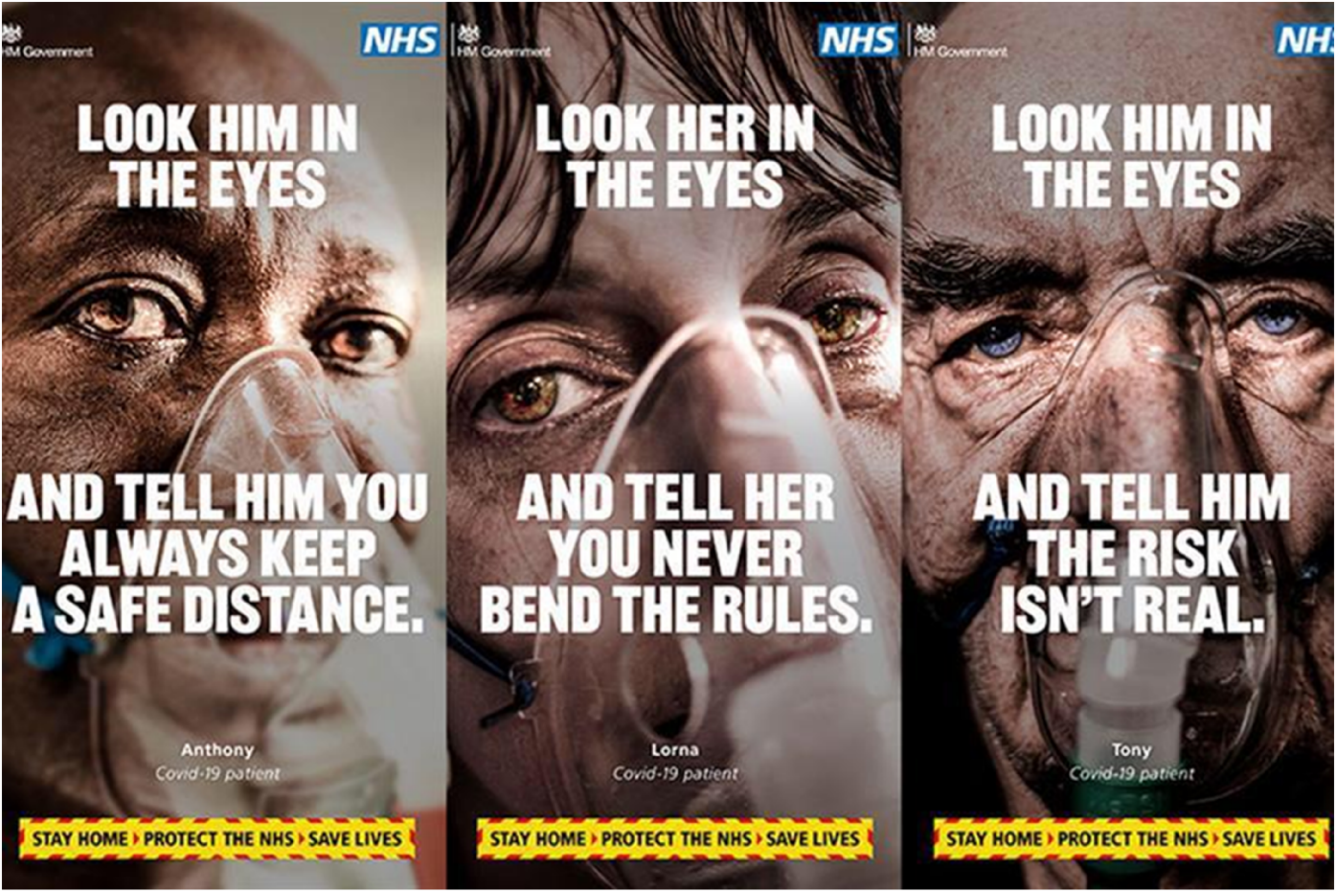
‘Look him / look her in the eyes’ (UK)

Source: Https://www.campaignlive.co.uk/article/will-governments-new-emotive-covid-ad-people-obey-rules/1705634

**Figure 3.**
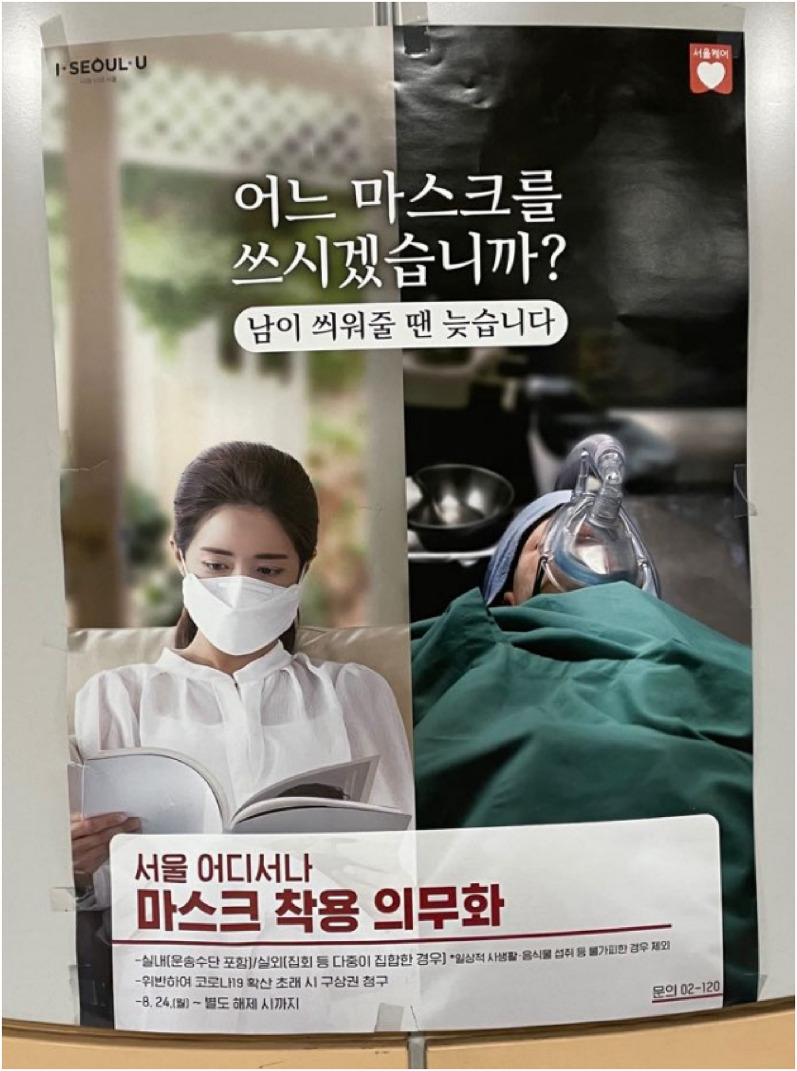
‘If someone puts it on for you, it's too late’ (South Korea)

The individualized address and top-down state-to-citizen commandment are especially vivid in an aggressive campaign launched by the UK government in February 2021 during the country's third lockdown (Figure 2). The campaign was based on a series of graphic images depicting sick people with ventilator breathing masks on their faces, demanding that the viewer ‘Look him’ or ‘Look her’ ‘in the eyes’ and commit to being an obedient responsible citizen by ‘always keep[ing] a safe distance’, ‘never bend[ing] the rules’ and ‘telling him the risk isn't real’ (a reprimand to Covid-19 deniers). Resembling an admonishing voice of a teacher or a parent, the campaign employs scare tactics that infantilize and self-responsibilize the viewer. Scare tactics are also evident in the South Korean government campaign, which shows a masked young woman flipping through a magazine (Figure 3). Adjacent to her in what appears to be a constricted space is a patient with a ventilator. The caption ominously reads: ‘If someone puts it on for you, it's too late.’ Another example, of a poster positioned at a South Korean subway turnstile, shows a masked cheery young couple with a caption echoing the same rationale for masking: ‘Mask up, for everyone's life and yours.’

**Figure 4.**
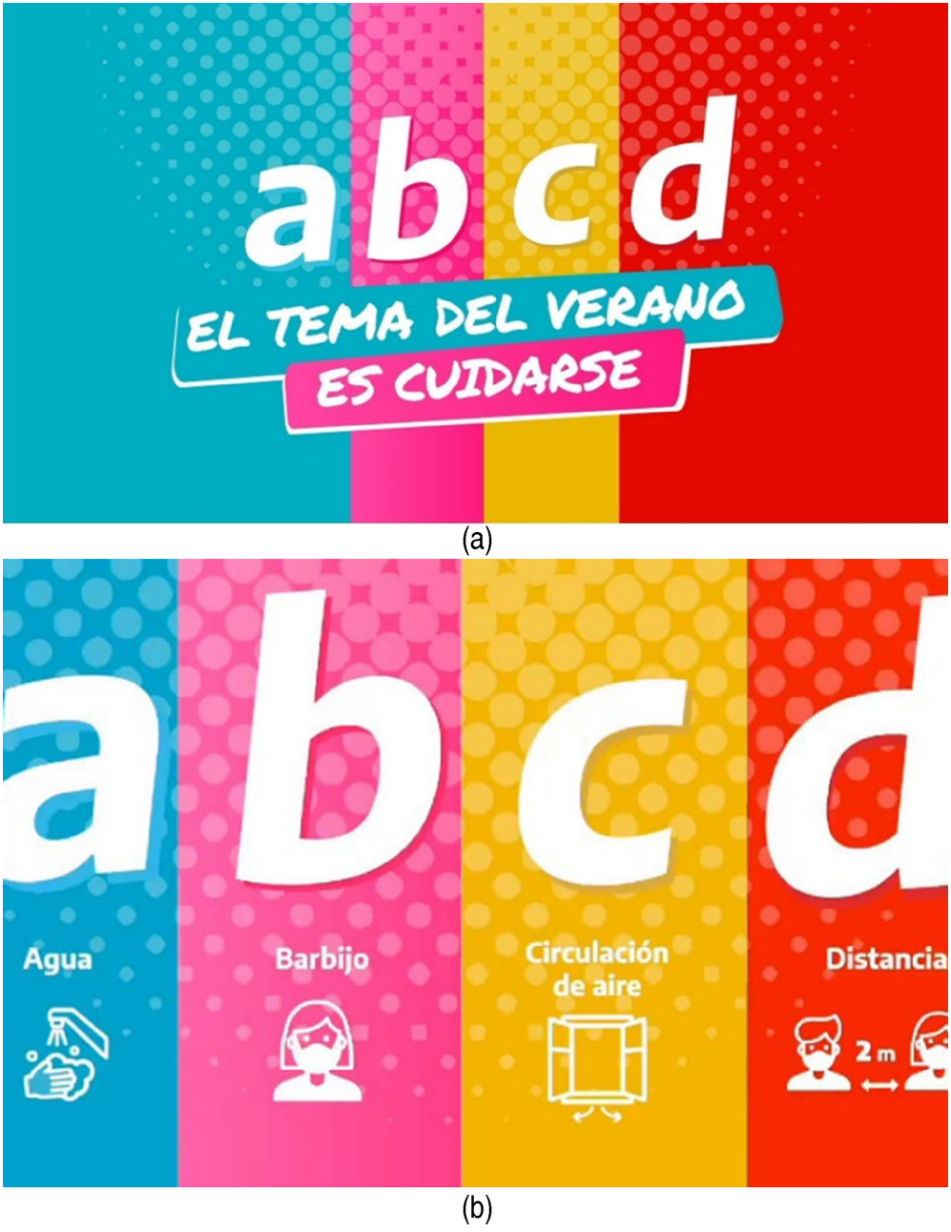
Two versions of ‘The ABCD for this summer’ (Argentina)

**Figure 5.**
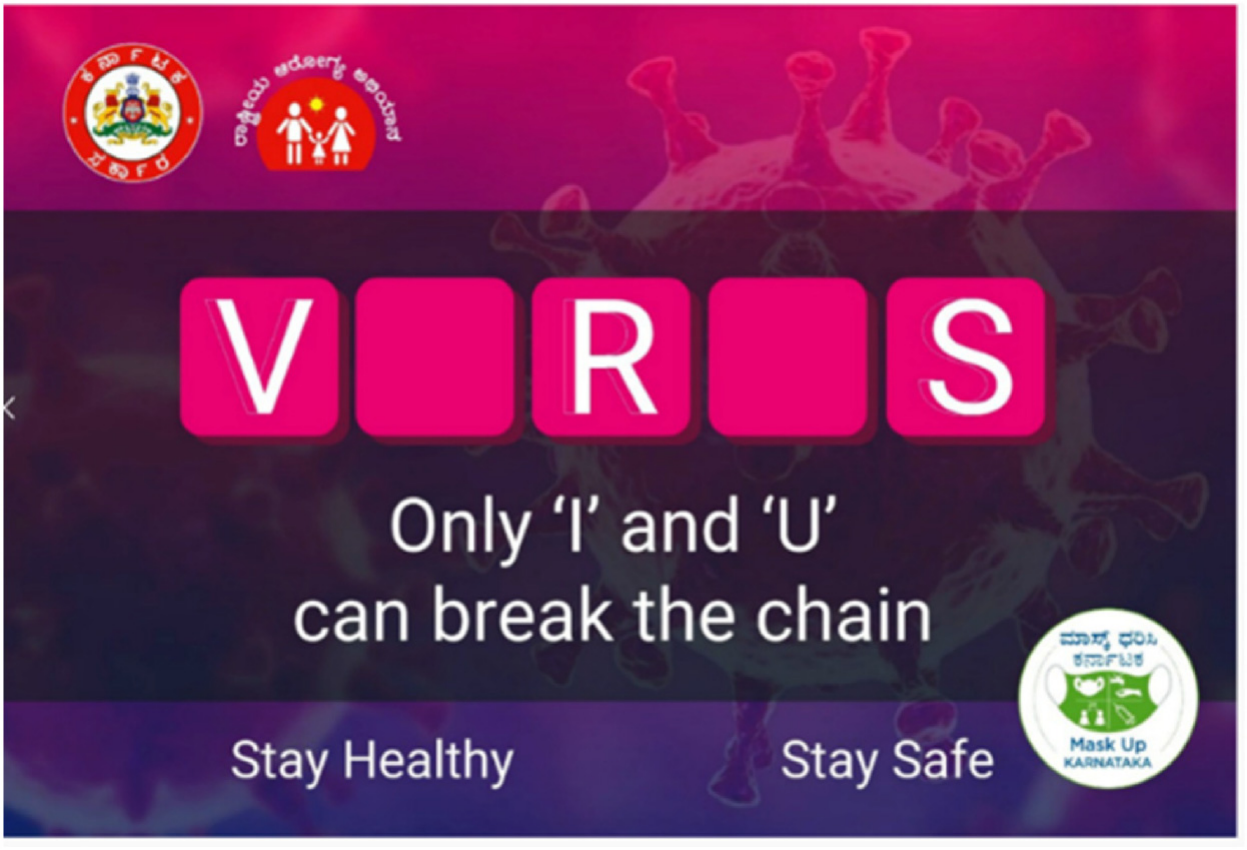
‘V_R_S: only ‘I’ and ‘U’ can break the chain’ (India)

Similar modes of paternalistic ‘nudging’ (Le Grand and New, 2015) are employed by other governments’ campaigns. For instance, the design of the Argentinian campaign resembles a poster used in classrooms to teach children the alphabet (Figure 4a and 4b). It displays the letters ABCD, each on a background of a different bright colour, accompanied by a key word and an illustration signifying ‘the ABCD for this summer’: ‘A: Agua’ (meaning water, with an illustration of washing hands); ‘B: Barbijo’ (meaning facemask, with an illustration of a woman wearing a face mask); ‘C: Circulación de aire’ (meaning air circulation, with an illustration of an open window and two arrows); and ‘D: Distancia’ (meaning distance, with an illustration of a man and a woman wearing face masks with an arrow indicating they are two metres apart). A similar infantilizing ‘educational’ approach can be seen in one of India's campaign posters produced by the state of Karnataka. It shows five Scrabble letter tiles, meant to make up the word ‘Virus’, with two letters missing: ‘V_R_S’. Underneath, the reader is given the answer: ‘Only “I” and “U” can break the chain’ (Figure 5).

These infantilizing, self-responsibilizing and sometimes punitive messages that have circulated during the pandemic, contrast starkly with the complete absence of any acknowledgement of government responsibility: the negligence and lack of timely interventions which exacerbated the crisis; the deprivation of care resources over decades of austerity and cuts; the impossible conditions of care workers; and the persistence of intersecting structural oppressions and socioeconomic disparities. As Dowling (2021: 189) observes, ‘the emphasis on personal responsibility … disregards both the interdependence of social relationships and the reality of structural inequalities that determine societal access to care as well as the distribution of care work.’

Indeed, amidst a devastating crisis of care, which the pandemic exposed and has intensified hugely, the national Covid-19 campaigns we examined advocate a ‘self-care fix’: putting personal responsibility centre stage, privatizing the responsibility for care and perpetuating the notion that ‘if you help yourself, then everyone will be helped’ (Dowling, 2021: 186). Variations of this self-care imperative can be found across many of the national campaigns, from Mexico's ‘Stay home. If you take care of yourself, we take care of us all’; through Spain's ‘Stopping COVID is a shared responsibility. If you protect yourself, you protect the others’; to Chile's ‘Taking care of each other is everyone's job’ and India's ‘Only “I” and “U” can break the chain’ and ‘I protect you, you protect me’. Like the post-2008 financial crisis branding narratives analysed by Banet-Weiser (2012), so too these Covid-19 national campaigns mobilize and authorize individual citizens to deal with the crisis on their own, mandating them to take care of themselves and each other.

The discrepancy between governments’ self-care and self-responsibility exhortations and the state's abdication of its duty to care for its citizens was thrown into sharp relief in Chile’s June 2020 national campaign. Resembling the UK campaign’s employment of scare tactics discussed earlier, the Chilean government launched an aggressive campaign under the slogan ‘You could be the next one.’ The campaign shows images of people in ambulances accompanied by the warning that ‘this would not happen if everyone respected the quarantine.’ In one of the ads an old woman is depicted as having contracted the virus following a visit from her grandchildren, who did not know they were infected. The campaign, which provoked fierce criticism from the public, privatizes the responsibility and puts the blame for spreading the virus entirely on individuals. Strikingly, it ignores a key structural factor: almost a third of the total Chilean workforce operates in the informal sector (OECD, 2021), thus considerable part of this workforce was unable to work from home and, inevitably, was at higher risk of infection. These workers were structurally unable to perform the ideal neoliberal subject demanded and idealized by the campaign.

Relatedly, the very capacity for self-care and, indeed, for survival, is inextricably linked to and dependent upon being a citizen. As Amaya (2015: 167) observes in another context, the hegemonic idea of a self-interested free will underpins the assumption of citizenship, and so ‘the pleasures of survival, success, accumulation, and mastery cannot be dissociated from the citizenship assumption’. Tellingly, there are no migrants or refugees – at least none are signified as such – in the campaigns. Thus, for example, the three people whose faces adorn the UK ‘Look him/her in the eyes’ posters and stand for Covid-19 patients in hospitals, are named ‘Anthony’, ‘Lorna’ and ‘Tony’ – all typical English names. In the Indian government's campaigns, the people portrayed all have light complexions and wear clothing suggestive of an urban middle class – a visual depiction that totally disregards entire, and arguably the most vulnerable, sections of the population.

**Figure 6.**
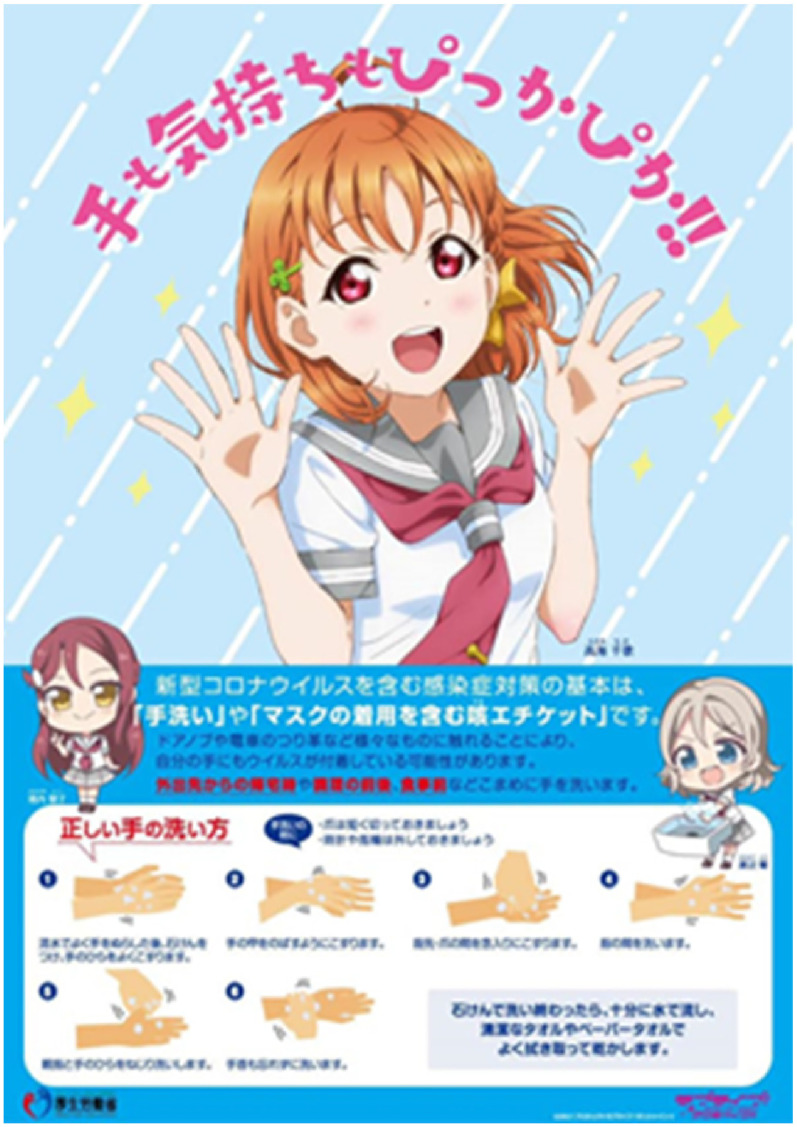
‘My hands and my feelings are shining/spotless’ (Japan)

The Covid-19 national campaigns in our sample idealize neoliberal citizens who not only govern their behaviour by obeying the rules, but also, crucially, govern their thinking and feelings. For instance, the Japanese campaign depicts a female cartoon figure in a happy pose with her palms wide open, saying ‘My hands and my *feelings* are shining/spotless’ (emphasis added) (Figure 6). Similarly, the UK's campaign, mentioned earlier, demands the viewer to ‘Look him’ or ‘Look her’ ‘in the eyes’: that is, following the rules is not sufficient on its own, it must be grounded in and derive from *feeling* accountable for the safety and the lives of others. In yet another example from one of the Indian campaigns in October 2020, when the pandemic was fairly under control, the government launched a Jan Andolan campaign encouraging citizens to register online and take an e-pledge to observe Covid-19 appropriate behaviour and get a certificate of commitment. Prime Minister Modi then tweeted that ‘India's COVID-19 fight is people driven and gets great strength from our COVID warriors’ (@narendramodi, 2020). The message here and in other campaigns is unequivocally that individuals ought to observe the rules and take it on themselves to save their fellow citizens.

**Figure 7.**
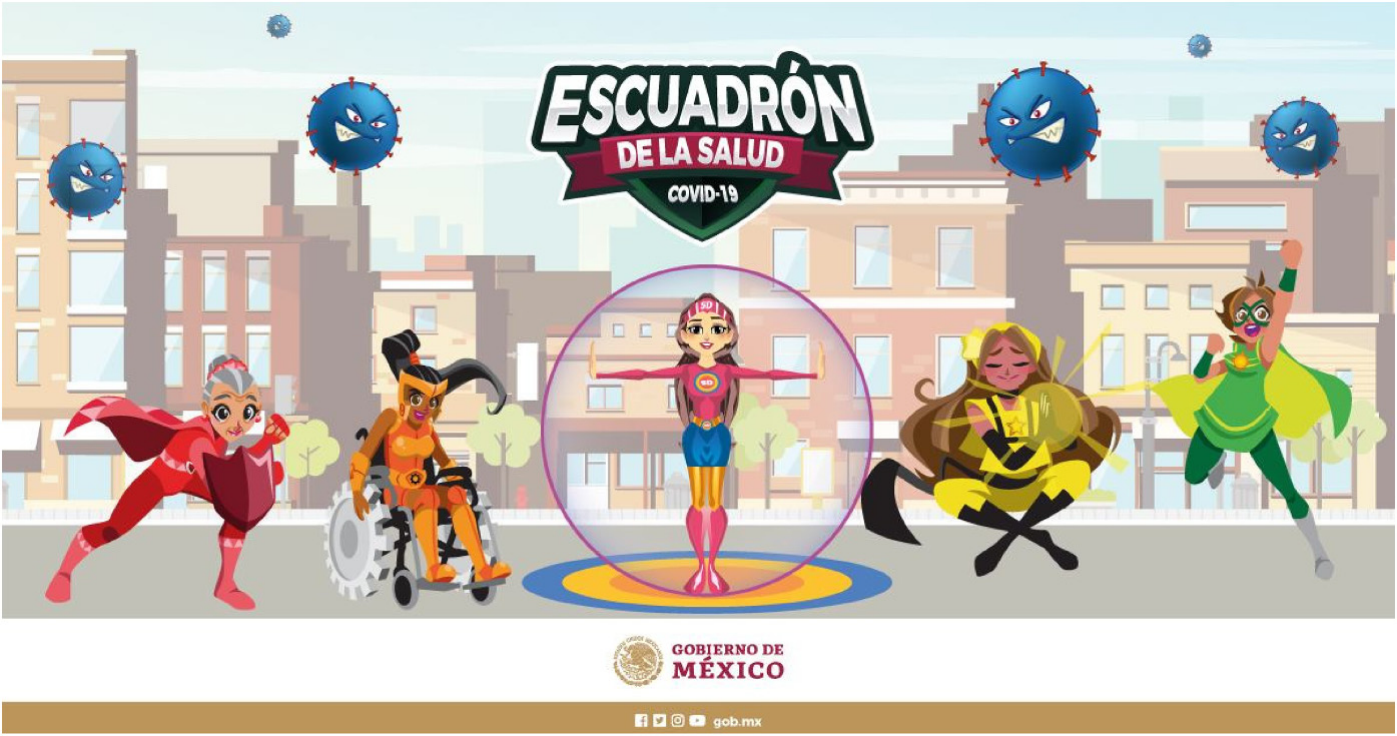
‘Escuadron de la salud covid-19’ (Mexico)

Source: Https://www.facebook.com/SaludDGPS/photos/las-cuatro-grandes-hero%C3%ADnas-que-junto-con-susana-distancia-formar%C3%A1n-el-escuadr%C3%B3n/10159846931439638/

Furthermore, the ideal neoliberal citizen evoked by the national campaigns is obliged to exercise both self-care *and* care for others. This is illustrated by a poster exhorting young Indian citizens to follow appropriate behaviours and encourage others to do the same. In it, the Indian government appropriates a quote attributed to Gandhi: ‘Be the change you wish to see in the world.’ The phrase, which suggests that all change begins and ends with the self, is a decontextualized and inaccurate paraphrasing of Gandhi's belief in the connection between personal and social transformation, deployed to motivate India's ‘Covid warriors’. Another example of the self-care and care-for-others imperatives can be found in the text (in both Russian and Kazakh) on a red background of an outdoor billboard ad of Kazakhstan's campaign: ‘YOU are responsible for *our* lives – wear a mask’ (Figure 1). However, nowhere is the casting of citizens as responsible for caring for – or indeed, saving – others more conspicuous than in Mexico's national campaign. Playing on the words ‘Su Sana Distancia’ which mean ‘healthy distance’, the campaign presents a cartoon super heroine figure, named ‘Susana Distancia’, who, together with her all-female ‘health squad’, fights against the evil virus (Figure 7). Strikingly, the cartoon female ‘health squad’ of self-reliant heroines was introduced by Mexico’s Health Ministry amidst a sharp rise in Covid-19-related deaths. Earlier in the pandemic, the Mexican government had been downplaying the threat from the virus and misreporting the number of cases and deaths, despite local officials repeatedly alerting it to the true numbers (Ahmed, 2020). Ironically, it is precisely as the state abdicates responsibility for handling the crisis and actively covers up its negligence, failures and lies, that individuals are called on to save the day.

Thus, instead of what Tronto (2013) calls a ‘caring democracy’, which is predicated on a politics that puts care at its left, right and centre (see also Briggs, 2017; Chatzidakis et al., 2020), the national Covid-19 campaigns promote a self-care democracy: one that relegates the fundamental care responsibilities to the individual and celebrates national solidarity ‘lite’, while the state's collective responsibility and the urgent need for redistributive policies are elided and disavowed.

## The gendered model of the crisis

During the pandemic, scientists have been busy ‘modelling’ development of the crisis, that is, producing mathematical models to understand how the virus might affect populations, in order to help inform government policy around the world. However, in this final section of our analysis we use the term ‘modelling’ in another way (but connected deliberately to its scientific meaning). We use ‘modelling’ as a way of interrogating the kinds of bodies and faces that *modelled* the pandemic in national government communications, and how these models shaped the imagination of the crisis. Who is the ideal self-responsible subject being called upon by the national Covid-19 campaigns? We noted several interesting common features in our sample. The most striking is related to the gendering of the figures used in these campaigns, and how these figures are mobilized to promote a national(ist) imaginary.

As mentioned earlier, most campaigns include illustrations or photographs of human characters in their ads. From a total of 45 images of human characters across the examples in our sample, 31 are of women, 12 are of men, and two are of children (a boy and a girl). Across all these examples, female figures are used to represent the caring, responsible citizen who follows the rules. For example, in Kazakhstan's national campaign, an image of a masked woman, displayed across billboards and public communications, became the most recognizable national face of the pandemic. Another example is a series of public messages under the banner ‘Help us to Help you'* (*#indiafightscorona*)*, in which the Indian government appeals to the public to ‘be smart’ and ‘be kind’ like the characters portrayed. The male character Uncle-ji (*ji* being an honorific), who occupies three posters in the series, is said to be checking facts before forwarding messages about Covid-19, paying his security guard full wages despite the lockdown and buying supplies and medicine for the family and his neighbour. He is portrayed as a superhero dressed with a cape, signifying control, and wearing glasses, signifying his intelligence (resembling somewhat the cinematic Clark Kent/Superman). The reference to employing a security guard indicates a level of urban affluence and a classed notion of domesticity which comes with a comfortable home, digitally connected social network and security. By contrast, in the other posters in the series, which depict female characters across different ages – a girl, Sneha, a young woman, Preeti, a middle-aged woman, Aunty-ji, and an old woman, Amma-ji – the women are seen in relational roles, taking care of their friends, staying indoors and helping with chores. The characters are assigned stereotypical gender roles of a caring daughter, grandmother, friend and neighbour, modelling the gendered ‘smart’ and ‘kind’ citizen the government exhorts them to be.

**Figure 8.**
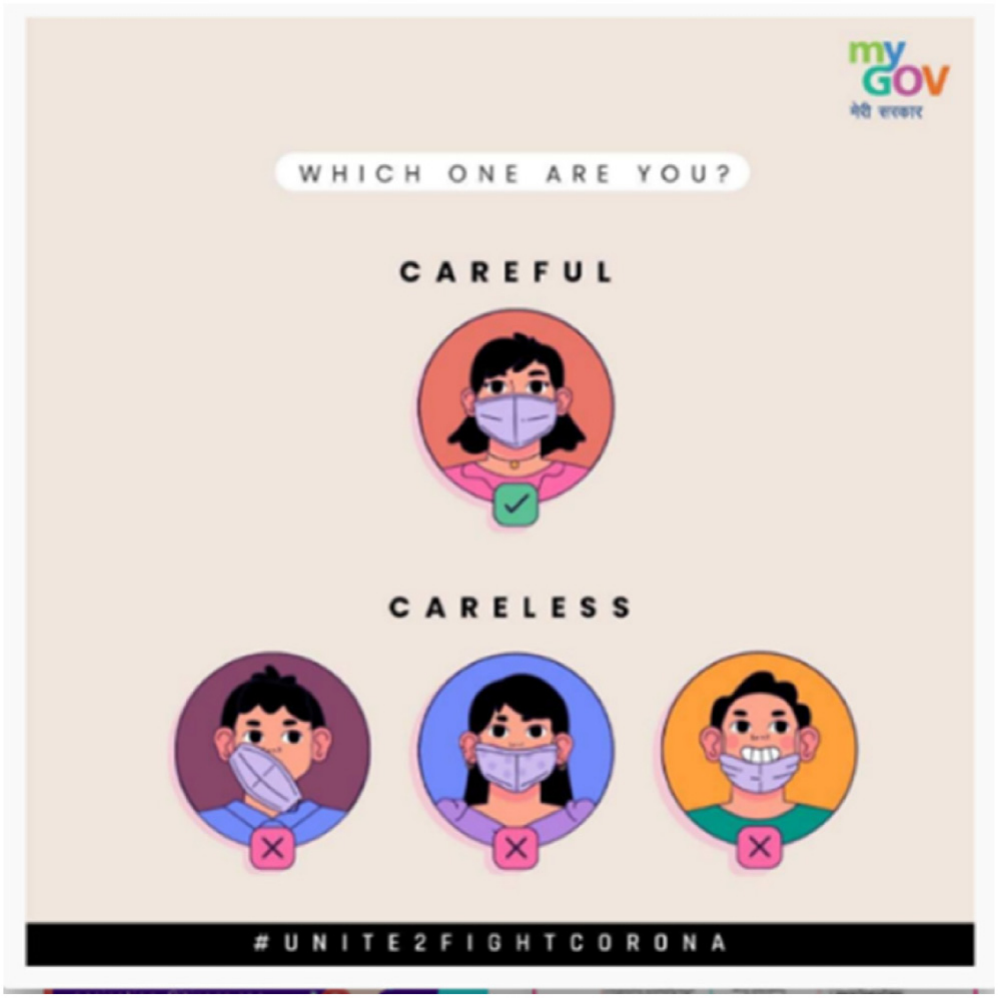
‘Which one are you? Careful / careless?’ (India)

In Japan, feminine *manga* characters are used to promote messages of hygiene and social distancing, evoking traditionally feminine traits of physical and affective cleanliness (see Figure 6). Female figures also dominate the UK campaign of April 2021, when lockdown was eased to allow up to six people from two households to socialize in parks and gardens and outdoor sports facilities to reopen. In three posters of this campaign, women embody the responsible and sensible citizen who remains cautious even as restrictions are being relaxed. For example, in one poster, a young woman – coded as middle class by the setting of her home and the private garden that can be seen from the window – is seen sitting smiling at her table with a laptop, a smartphone, and a mug of coffee on it. The image connotes familiar stock images of entrepreneurial female workers, and the text confirms her belonging to a privileged sector of workers who can carry on their waged work from home: ‘Thinking of going in? Keep working from home if you can.’ In India, images of women – many depicted as mothers pictured with their children – dominate the campaign's ads, representing the model responsible citizen who takes care not only of herself but also of others. For example, in an ad (Figure 8) which is part of the national #UNITE2FIGHTCORONA, the text asks the viewer: ‘Which one are you?’ and offers two responses: ‘Careful’ – exemplified by an illustration of a woman wearing a mask that properly covers her chin and nose, and ‘Careless’ – illustrated by three images of people (two men and one woman) wearing their masks incorrectly. Even among the figures representing the ‘careless’, the woman appears the least careless; the men's masks are conspicuously misplaced while the woman's mask fails only to cover her small nose. In another of India's Government of Karnataka posters, a photograph of a young masked woman is accompanied by text in quotation marks (signifying her voice) which reads: ‘Our life is our responsibility; wear a mask, maintain social distance.’ The voice of the state is merged into and presented as the voice of the individual woman.

These constructions notably draw on the historical trope that constructs women in times of crisis as guarantors of the social order and emphasizes their responsibility and accountability in periods of great unrest (Lawler, 2000). The gendering of the responsible citizen draws on longer narratives of feminizing the nation and naturalizing women's roles as social reproducers of the nation (Thomson, 2020; Yuval-Davis, 1997). The campaigns analysed here illustrate, yet again, how gendered roles and narratives are summoned to consolidate the national project during times of crises. For example, in Europe and the US, women, and especially mothers have been constructed as both the cure and the cause of socioeconomic crises (Gillies, 2007; Lawler, 2000), while austerity discourses in the UK have consistently interpellated a feminine subject position (Bramall, 2013), often casting women as personal respondents to the economic downturn and stressing their responsibility and need for positive thinking (Negra and Tasker, 2014). These representations concurrently capitalize on the contemporary postfeminist trope that positions women as ideal neoliberal subjects: self-reliant individuals who can lead responsibilized and self-managed lives through self-improvement and self-transformation (Gill and Scharff, 2011; McRobbie, 2009).

Echoing these historical and contemporary tropes, the female figure in Covid-19 national campaigns models an ideal citizen who exercises self-responsibility and self-governance while simultaneously taking active responsibility for her community and the nation. This dual female responsibility is vividly exemplified by one of the Israeli campaign's posters, which is fronted by an image of a young female business owner in a red shirt and black jacket, her hair down, as she looks towards the horizon beyond the photo’s frame. Capitalizing on familiar postfeminist stock images of assertive and ambitious career women, the poster casts the woman in the role of the responsible worker who strives to get both her personal finances *and *the national economy back on track. ‘The business has been closed for two months; I’m not taking any chances!’, she is quoted as saying, beneath which the national slogan ‘We will be fine OR we will be in lockdown’ is presented in a bolded frame and capital letters. Resembling the example from India’s (Karnataka’s) posters discussed earlier, here too the personal and the national voice converge in a single message delivered by the ideal female citizen. Thus, in many of the campaigns, women embody the nation itself who adjures its citizens/children to behave responsibly.

Similar popular postfeminist ideas of capacity and empowerment to that observed in other campaigns (Israel; UK) also animate the Mexican campaign mentioned earlier, with its all-female super heroine ‘health squad’ (Figure 7). The cartoon health squad includes five characters, four of whom represent the colours of the national Covid traffic light system to help the public to understand the pandemic rules. The older woman, Refugio, signified by her grey hair, represents the colour red, which means ‘Stay home’, while Prudence, a woman in a wheelchair, represents orange and its message ‘Avoid leaving home’. Esperanza, identified as a ‘muxhe gunna’ – someone whose female identity is the same as the sex she was assigned at birth – represents yellow and exhorts obedience to health measures when going out. Aurora, who represents green, is queer and her superpower is the 'new normal'. The fifth super heroine is the squad's leader, Susana Distancia, a slim feminine figure, with long brown hair, dressed in a tight pink shirt and a blue miniskirt.

Notably, the Mexican campaign, which is designed deliberately as a ‘diversity ad’ and is part of contemporary ‘intersectional femvertising’ (Kanai and Gill, 2020), leaves men entirely out of the narrative and off the hook. Mexico's male-dominated government, led by President Andrés Manuel López Obrador, has repeatedly been accused of sexism and policies that disadvantage women (Webber, 2021). At the start of the pandemic, a shocking 73% of women in Mexico lost their jobs (Webber, 2021) and, despite a sharp rise in gender-based violence since the introduction of lockdown measures, the Mexican government announced that, as part of an emergency decree, it was slashing funding for women's services and redirecting money to programmes it considered having greater priority (Equality Now, 2020). Against this careless masculine government (whose austerity programmes were already in place before the pandemic), whose responsibility for its citizens is not even hinted at in the national campaign, the animated all-female ‘health squad’ is fully responsibilized for saving the nation.

More broadly, across all the examples we analysed, none of the campaigns mentions explicitly or hints at the multiple devastating effects of the pandemic that have hit women across the world disproportionately, including the dramatically unequal impact of home-schooling on women, the spiralling rates of domestic violence, the devastating economic losses suffered by women, and the severe impact on women's mental and physical health. These realities are crudely disavowed, as are the structures that underpin them, whose addressing is primarily the responsibility of the state. Across these campaigns, women are positioned as responsible, resilient and caring individuals, embodying the nation's ideal warrior citizen.^4^

## Conclusion

Our analysis of national governments’ Covid-19 campaigns reveals the circulatory power of a market-driven neoliberal logic that underpins national responses to the ongoing pandemic crisis. The pandemic has presented national governments with unique conditions for articulating and mutually reinforcing nationalism and neoliberalism. Capitalizing on individuals’ and communities’ vulnerability and dependence on the state to provide social and health protection, national government campaigns appropriated discourses of collectivity, solidarity, unity, mutual support, and collective care, to centre the nation and reinforce an imagined egalitarian national community. At the same time, they hollowed out these discourses of their welfarist context, mobilizing them instead to direct responsibility on to the individual, absolve the state of its social care responsibilities, and consolidate an image of the national public as an atomized aggregate of responsible individuals. Thus, the campaigns work to cement a neoliberal rationality, but they do so under the veneer of national solidarity and egalitarian collectivity. Rather than the blatant abjection of welfarism and the racialized and classed denigration of dependence and vulnerability that have intensified and been normalized in public discourse since the 1980s (Briggs, 2017; Chatzidakis et al., 2020; McRobbie, 2020), the national Covid-19 campaigns we examined seemingly celebrate interdependence, but an interdependence whose essence, paradoxically, is self-care; as encapsulated by one of the Mexican slogans: ‘If you take care of yourself, we take care of us all.’

While our small sample is by no means representative of the large number of national campaigns that have been produced during the pandemic, some striking patterns are notable. First, across the campaigns, there is a consistent national imperative to co-opt citizens to become Covid-19 warriors ever ready to save themselves and live up to the spurious and recurring anthem: ‘Save yourself to save others'. In appealing to individuals to adhere to the behavioural scripts required to stave off contagion, the campaigns efface the role and responsibility of the state at a time of crisis. Instead, the state assumes a stance of a benevolent parent urging family members – and specifically women – to take care of one another. The campaigns deflect attention from the level of the collective and public to the individual and the realm of the private, and re-inscribe gendered notions of responsibility, care and citizenship through casting women in the role of the nation's ideal self-responsibilized citizen-warrior.

Second, while clearly our lives and politics are globally entangled and interdependent, and this entanglement is perhaps nowhere more pronounced than in times of a global pandemic, in the national campaigns there is a noticeable absence of a world outside the nation. Rather, they encourage a turning inwards and a disavowal of any sense of interdependence between and across borders, nations, and bodies. Furthermore, with very few exceptions, the national Covid-19 campaigns assume an able-bodied citizen with a rooted sense of territoriality, domestic stability and equally distributed infrastructural access. Where are the bodies of transient populations or the unhoused, or bodies that lack the ability to practise these repertoires of safety, hygiene and self-responsibility? With the staggering numbers of deaths, and of severely ill and displaced people across the globe, this insistence on the national individualized self is manipulative and furthers the global erasure and elision of vulnerable bodies (Hegde, 2020).

The campaign messages appear to appeal to everyday intuitive knowledge about cleanliness, care and consideration for our neighbours, as exemplified by governments’ relentless appeal to the public to use their ‘common sense’ (e.g. Denmark, Spain, UK). Yet, as we have shown in this article, these messages simultaneously perform deeper – and deeply problematic – ideological work. What politicians are really doing when they appeal to common sense, Hall and O’Shea (2013) observe, is shaping popular opinion. Indeed, capitalizing on citizens’ state of crisis-readiness in the contemporary hyper-mediated environment (Frosh and Pinchevski, 2009), national government communications have constructed the Covid-19 pandemic as a crisis to which the commonsensical response is resolutely individualized, self-responsibilized and gendered.

In other domains and contexts, the pandemic might have encouraged a process of reckoning; perhaps even a crisis of dominant narratives, especially in relation to the urgent need to address structural inequalities, redistribute resources and to value and invest in social care. However, in government national communications – at least the ones we examined in this article – the crisis has been used to revitalize and cement a national neoliberal narrative. It is partly through this narrative that governments have rendered the pandemic legible as a crisis, while it is also through this very narrative that they have rendered it illegible; that is, they have obscured and disavowed the structural injustices that underpin and exacerbate the crisis, and, crucially, the structural changes required to address it.

## Notes

1 Given the huge discrepancies between vaccination rollouts in different countries, we decided to exclude communications about vaccinations from our sample.

2 The sample breakdown is as follows:

1. Argentina – two posters from the ‘ABCD’ campaign;
2. Chile – three online texts (‘Let's take care of each other’; ‘Taking care of each other is everyone's job’; ‘You could be the next one’);
3. Denmark – one online text (‘Apart, to get together again’);
4. India – 15 posters: 2 from the #UniteToFightCorona campaign, 4 from the #KarnatakaFightsCorona campaign, 1 from the MyGov campaign, 2 from the #TogetherAgainstCOVID19 campaign; 1 online poster from the Fight Covid-19 with Yoga campaign; and 5 posters from the #IndiaFightsCorona campaign;
5. Israel – one online poster from the ‘it will be fine/we will be in lockdown’ campaign;
6. Japan – two posters: ‘My hands and my feelings shine/ are spotless’ and ‘Together we stop the spread’;
7. Kazakhstan – two billboard ads: ‘YOU are responsible for our lives – wear a mask! COVID-19’ and ‘Wear a mask. Let's get back to old ways of life’;
8. Mexico – one online poster with the national slogan ‘Stay home. If you take care of yourself, we take care of us all’; and four online images from the Susana Distancia campaign;
9. South Korea – two billboard ads, one stating ‘wear a mask’ and another ‘Limit conversations during your meal’ (Ministry of Food and Drug Safety); and a poster showing a patient on a ventilator with the message ‘When someone puts it on for you it's too late’ (Seoul Metropolitan Government).
10. Spain – one poster with the slogan ‘We stop this virus together’;
11. Switzerland – one billboard ad with ‘New Coronavirus: This is how we protect ourselves’ slogan;
12. UK – a Gov.uk online poster ‘Hands. Face Space’ campaign; three posters from the ‘Look Him/Look Her in the Eyes’ campaign; and three billboard posters from the ‘Let's take this next step safely’ campaign.

3 Meme 41 on https://thetab.com/uk/2020/05/11/stay-alert-government-lockdown-message-memes-155878

4 Gill and Orgad (2022) observe a related paradox in the advertising and lifestyle coaching that circulated during the pandemic, whereby ‘at precisely a moment when structural inequalities and injustices become so visible in media, women are insistently interpellated with individualistic, psychologised forms of address’.
